# Linac-based stereotactic radiosurgery for brain metastases: Survival and salvage treatment in a contemporary cohort of 3893 patients

**DOI:** 10.1093/noajnl/vdag242

**Published:** 2026-09-16

**Authors:** Drew T Bergman, Mitchell I Parker, Henry Walch, Åse Ballangrud, Michalis Aristophanous, Melissa Zinovoy, Brandon S Imber, Gustav Cederquist, Christopher Jackson, Connor J Kinslow, Anthony Abaya, John J Cuaron, James Janopaul-Naylor, Atif Khan, Zachary R Moore, Boris A Mueller, Jacob Y Shin, Divya Yerramilli, Yoshiya Yamada, Yao Yu, Luke R G Pike

**Affiliations:** Department of Radiation Oncology, Memorial Sloan Kettering Cancer Center, New York, New York, USA; Department of Radiation Oncology and Applied Sciences, Dartmouth Cancer Center, Lebanon, New Hampshire, USA; Department of Radiation Oncology, Memorial Sloan Kettering Cancer Center, New York, New York, USA; Department of Epidemiology and Biostatistics, Memorial Sloan Kettering Cancer Center, New York, New York, USA; Department of Medical Physics, Memorial Sloan Kettering Cancer Center, New York, New York, USA; Department of Medical Physics, Memorial Sloan Kettering Cancer Center, New York, New York, USA; Department of Radiation Oncology, Memorial Sloan Kettering Cancer Center, New York, New York, USA; Department of Radiation Oncology, Memorial Sloan Kettering Cancer Center, New York, New York, USA; Department of Radiation Oncology, Memorial Sloan Kettering Cancer Center, New York, New York, USA; Department of Radiation Oncology, Memorial Sloan Kettering Cancer Center, New York, New York, USA; Department of Radiation Oncology, Memorial Sloan Kettering Cancer Center, New York, New York, USA; Department of Medical Physics, Memorial Sloan Kettering Cancer Center, New York, New York, USA; Department of Radiation Oncology, Memorial Sloan Kettering Cancer Center, New York, New York, USA; Department of Radiation Oncology, Memorial Sloan Kettering Cancer Center, New York, New York, USA; Department of Radiation Oncology, Memorial Sloan Kettering Cancer Center, New York, New York, USA; Department of Radiation Oncology, Memorial Sloan Kettering Cancer Center, New York, New York, USA; Department of Radiation Oncology, Memorial Sloan Kettering Cancer Center, New York, New York, USA; Department of Radiation Oncology, Memorial Sloan Kettering Cancer Center, New York, New York, USA; Department of Radiation Oncology, Memorial Sloan Kettering Cancer Center, New York, New York, USA; Department of Radiation Oncology, Memorial Sloan Kettering Cancer Center, New York, New York, USA; Department of Radiation Oncology, Memorial Sloan Kettering Cancer Center, New York, New York, USA; Department of Radiation Oncology, Memorial Sloan Kettering Cancer Center, New York, New York, USA

**Keywords:** brain metastases, linac-based SRS, overall survival, stereotactic radiosurgery, whole-brain radiation therapy

## Abstract

**Background:**

Real-world outcomes after stereotactic radiosurgery (SRS) for brain metastases across the full burden spectrum are limited. We tested whether overall survival (OS) in higher-burden lesion groups is non-inferior to a 5-10 lesion reference and characterized salvage patterns, including whole-brain radiotherapy (WBRT).

**Methods:**

A total of 3893 consecutive patients received linac-based SRS (1-5 fractions) at seven sites of an NCI-designated cancer center network (2017-2026), stratified by lesion count (1, 2-4, 5-10, 11-20, >20). Multivariable Cox regression used 5-10 as reference; pre-specified non-inferiority testing (margin HR 1.30, one-sided α = 0.025) compared higher-burden groups. Cumulative incidences of salvage interventions used death as the competing event.

**Results:**

Median age 65 years; median follow-up 41.2 months. 77% had 1-4, 17% had 5-10, and 6% had >10 lesions (maximum 140). Median OS was 16.2, 11.0, 8.2, 8.7, and 6.5 months for the 1, 2-4, 5-10, 11-20, and >20 groups. OS was significantly better for 1 (HR 0.62, 0.55-0.69) and 2-4 (HR 0.81, 0.73-0.90) versus 5-10; non-inferiority was met for 11-20 (*P* < .001) but not >20 (*n* = 41, *P* = .20). CNS-directed systemic therapy was strongly protective (HR 0.62, 0.57-0.68); 21.7% of patients with ≤20 lesions survived ≥30 months. 12-month salvage WBRT incidence was 2.9%-6.3%; 24-month incidence was 6.7% versus 41.7% for a third SRS course.

**Conclusions:**

OS plateaued from 5 through 20 lesions, with non-inferiority to the 5-10 reference established for 11-20; the >20 group (*n* = 41) was underpowered and showed a directionally worse estimate that could not exclude a clinically meaningful decrement. Salvage WBRT was uncommon across all lesion-count groups (24-month cumulative incidence ≤7.3%; >90% of patients received no salvage WBRT), even after multiple SRS courses. These data support an SRS-first strategy guided by clinical factors rather than fixed lesion-count thresholds.

Key Points

- OS plateaued from 5 to 20 lesions (non-inferiority met for 11-20); the >20 group was underpowered. 1-4 lesions had better OS.
- Salvage WBRT was uncommon (24-month cumulative incidence ≤7.3%) across all lesion counts but rose with burden; treatment era independently improved OS.
- SRS candidacy should follow clinical factors, not fixed lesion-count cutoffs.

Importance of the Study

Contemporary guidelines increasingly endorse stereotactic radiosurgery over whole-brain radiotherapy for brain metastases, yet real-world survival and salvage data spanning higher lesion counts remain limited. In 3893 patients treated with linac-based SRS across the full intracranial-burden spectrum at seven sites of an NCI-designated comprehensive cancer center network, overall survival was similar between patients with 5-10, 11-20, and >20 treated lesions, and salvage whole-brain radiotherapy (WBRT) was uncommon across the burden spectrum (24-month cumulative incidence ≤7.3%), though it increased significantly with lesion number. Treatment era independently predicted improved survival and reduced WBRT utilization, reflecting the real-world impact of CNS-penetrant systemic therapies. These findings provide contemporary benchmarks to inform clinical decision-making and support extending stereotactic radiosurgery (SRS) to patients with high lesion counts without a rigid numerical threshold.

Brain metastases affect up to 30% of patients with solid tumors and are a leading cause of cancer-related morbidity and mortality.^1^ Whole-brain radiotherapy (WBRT) was the long-time standard of care, but it carries well-documented risks of irreversible neurocognitive decline that substantially impair quality of life.^2^ Linear accelerator (linac)-based frameless stereotactic radiosurgery (SRS) with single-isocenter volumetric-modulated arc therapy (VMAT) planning has transformed this paradigm, enabling noninvasive, precise, ablative, and rapid treatment of multiple metastases in a single session while sparing normal brain.

Randomized trials established the superiority of SRS over WBRT for neurocognitive preservation, functional independence, and quality of life, with equivalent survival, initially in patients with 1-4 lesions.^3-5^ The JLGK0901 prospective observational study extended the paradigm to ≤10 lesions,^6^ and a recent trial demonstrated superior quality of life for SRS versus hippocampal-avoidance WBRT with equivalent survival in patients with 5-20 lesions.^7^ Contemporary guidelines now endorse SRS across a broader range of intracranial burden, yet outcome benchmarks for >10, and especially >20, lesions remain scarce, and most prior evidence pre-dates the CNS-penetrant systemic-therapy era.^8^

Technical advances in linac delivery, robotic couches, improved geometric accuracy, high-definition multi-leaf collimators, and real-time intrafraction motion management now enable simultaneous treatment of dozens of lesions in a single session.^9^ At our institution, single-session, frameless SRS to >100 lesions has been delivered within a single standard treatment appointment, an approach that would be impractical with frame-based Gamma Knife platforms requiring multi-day fractionation for comparable burden. Survival and salvage data for these very high-burden groups in routine practice are sparse, limiting evidence-based counseling and trial design.

Here we report OS and salvage treatment patterns from the largest contemporary linac-based SRS registry to date, 3893 patients across 5 lesion-burden categories treated at 7 geographic sites of an NCI-designated comprehensive cancer center network from 2017 through 2026. We characterize the relationship between intracranial burden and survival in a cohort treated with contemporary systemic therapy, quantify cumulative incidences of salvage interventions and the degree to which WBRT can be deferred, identify independent predictors of salvage WBRT, and provide contemporary real-world benchmarks for the field.

## Methods

### Study Design and Cohort

This retrospective cohort study was approved by the institutional review board with a waiver of informed consent. We identified all patients aged ≥18 years who received linac-based SRS (1-5 fractions) for brain metastases at any of 7 institutional sites between January 2017 and February 2026 by systematic query of radiation oncology treatment planning and electronic health record databases. The database was locked for analysis on April 25, 2026. KPS, extracranial metastatic disease, and largest treated lesion diameter, incompletely documented in institutional databases, were coded as ‘Unknown’ to preserve the full analytic sample rather than restricting to complete cases. Active CNS-directed systemic therapy was derived from medication prescription records (see below) and treated as a binary variable. Patients without lesion-count data were excluded. The first SRS course per patient was the index course, and the end of treatment was time zero.

### Radiosurgery Technique and Planning

All treatments were delivered on Varian TrueBeam linear accelerators using frameless, mask-immobilized VMAT with cone-beam CT image guidance and intrafraction motion management. A single isocenter was used for most courses, with multiple isocenters employed when treating higher numbers of lesions or lesions prescribed different fractionation schedules. Target volumes were delineated on contrast-enhanced MRI fused to the planning CT, with a standard 1-mm planning target volume margin. Dose and fractionation were individualized according to lesion size, location, and prior radiotherapy; the three predominant regimens were 27 Gy in 3 fractions, 30 Gy in 5 fractions, and 21 Gy in 1 fraction.

### Lesion Burden Categorization

Index lesion counts were drawn from the institutional treatment-planning database. Patients were stratified into 5 groups: 1, 2-4, 5-10, 11-20, and >20 lesions. For competing-risk and Fine-Gray analyses, lesion burden was condensed to three groups (1, 2-4, ≥5 lesions).

### Endpoints

The primary endpoint was overall survival (OS) from the index SRS course to death from any cause, censored at last vital-status contact. Secondary endpoints were the cumulative incidences of (1) additional SRS (any subsequent SRS course, whether to a previously treated or new lesion), (2) salvage WBRT, (3) salvage craniotomy, and (4) any next CNS intervention, a composite defined as the first of these 3 salvage interventions to occur, each analyzed with death as the competing event.

### CNS Active Systemic Therapy

Active CNS-directed systemic therapy was defined as receipt of a systemic agent with documented intracranial response rates of >30% in published brain-metastasis trials. The pre-specified agent list comprised checkpoint inhibitors (eg nivolumab, ipilimumab); EGFR inhibitors (osimertinib, dacomitinib); ALK inhibitors (ceritinib, alectinib, brigatinib, lorlatinib); ROS1/NTRK inhibitors (entrectinib, repotrectinib); RET inhibitors (selpercatinib, pralsetinib); BRAF/MEK inhibitors (dabrafenib, trametinib); the multi-tyrosine-kinase inhibitor cabozantinib; the HER2-directed agents tucatinib and fam-trastuzumab deruxtecan; the CDK4/6 inhibitor abemaciclib; and capecitabine. Bevacizumab was not included on the active CNS-directed agent list. For descriptive distributions in Table 1, agents were grouped as immunotherapy (checkpoint inhibitors), targeted therapy (TKIs, BRAF/MEK inhibitors, and HER2-directed agents), or chemotherapy (capecitabine). For each patient, prescription records were collapsed to one row per (patient, agent), and a patient was classified as receiving active CNS-directed systemic therapy if at least one qualifying agent had a duration on drug of ≥30 days and remained active on or after the date of the index SRS course minus 30 days (ie the agent was being administered from approximately 1 month before the index SRS course onward).

**Table 1.** Patient and treatment characteristics by lesion count group

	Overall (*N* = 3893)	Lesion count group at first SRS course		
Characteristic	Overall	1 Lesion	2-4 Lesions	≥5 Lesions
N	3893	1641	1351	901
Age at SRS, median (IQR), years	65.0 (55.0-73.0)	65.0 (56.0-73.0)	65.0 (55.0-73.0)	63.0 (55.0-71.0)
**Age group**				
<70	2568 (66.0%)	1058 (64.5%)	882 (65.3%)	628 (69.7%)
≥70	1325 (34.0%)	583 (35.5%)	469 (34.7%)	273 (30.3%)
**Sex**				
Female	2272 (58.4%)	918 (55.9%)	791 (58.5%)	563 (62.5%)
Male	1621 (41.6%)	723 (44.1%)	560 (41.5%)	338 (37.5%)
**Primary cancer type**				
NSCLC	1301 (33.4%)	529 (32.2%)	464 (34.3%)	308 (34.2%)
Breast	640 (16.4%)	252 (15.4%)	207 (15.3%)	181 (20.1%)
GI	375 (9.6%)	174 (10.6%)	131 (9.7%)	70 (7.8%)
Melanoma	342 (8.8%)	119 (7.3%)	133 (9.8%)	90 (10.0%)
Other	1235 (31.7%)	567 (34.6%)	416 (30.8%)	252 (28.0%)
**Fractionation**				
Single-fraction	1161 (29.8%)	550 (33.5%)	421 (31.2%)	190 (21.1%)
Multi-fraction	2732 (70.2%)	1091 (66.5%)	930 (68.8%)	711 (78.9%)
**Post-operative SRS**				
No	3197 (82.1%)	1286 (78.4%)	1119 (82.8%)	792 (87.9%)
Yes	696 (17.9%)	355 (21.6%)	232 (17.2%)	109 (12.1%)
**Prior WBRT**				
No	3774 (96.9%)	1602 (97.6%)	1312 (97.1%)	860 (95.4%)
Yes	119 (3.1%)	39 (2.4%)	39 (2.9%)	41 (4.6%)
**Prior craniotomy**				
No	3133 (80.5%)	1257 (76.6%)	1095 (81.1%)	781 (86.7%)
Yes	760 (19.5%)	384 (23.4%)	256 (18.9%)	120 (13.3%)
**Treatment era**				
2017-2019	836 (21.5%)	377 (23.0%)	310 (22.9%)	149 (16.5%)
2020-2022	1323 (34.0%)	587 (35.8%)	442 (32.7%)	294 (32.6%)
2023-2026	1734 (44.5%)	677 (41.3%)	599 (44.3%)	458 (50.8%)
**KPS**				
≥90	1120 (28.8%)	528 (32.2%)	370 (27.4%)	222 (24.6%)
70-80	1186 (30.5%)	509 (31.0%)	406 (30.1%)	271 (30.1%)
<70	144 (3.7%)	55 (3.4%)	51 (3.8%)	38 (4.2%)
Unknown	1443 (37.1%)	549 (33.5%)	524 (38.8%)	370 (41.1%)
**ECM at BM diagnosis**				
No	308 (7.9%)	181 (11.0%)	90 (6.7%)	37 (4.1%)
Yes	3450 (88.6%)	1398 (85.2%)	1219 (90.2%)	833 (92.5%)
Unknown	135 (3.5%)	62 (3.8%)	42 (3.1%)	31 (3.4%)
**Any CNS-active systemic agent**				
No	2592 (66.6%)	1111 (67.7%)	903 (66.8%)	578 (64.2%)
Yes	1301 (33.4%)	530 (32.3%)	448 (33.2%)	323 (35.8%)
**Immunotherapy**				
No	3562 (91.5%)	1497 (91.2%)	1238 (91.6%)	827 (91.8%)
Yes	331 (8.5%)	144 (8.8%)	113 (8.4%)	74 (8.2%)
**Targeted**				
No	3250 (83.5%)	1381 (84.2%)	1122 (83.0%)	747 (82.9%)
Yes	643 (16.5%)	260 (15.8%)	229 (17.0%)	154 (17.1%)
**Chemotherapy**				
No	3491 (89.7%)	1478 (90.1%)	1220 (90.3%)	793 (88.0%)
Yes	402 (10.3%)	163 (9.9%)	131 (9.7%)	108 (12.0%)
**Largest lesion diameter**				
≤20 mm	2162 (55.5%)	780 (47.5%)	821 (60.8%)	561 (62.3%)
>20 mm	754 (19.4%)	255 (15.5%)	285 (21.1%)	214 (23.8%)
Unknown	977 (25.1%)	606 (36.9%)	245 (18.1%)	126 (14.0%)

### Statistical Analysis

OS was estimated by Kaplan-Meier and groups compared by log-rank. Median follow-up was estimated by reverse Kaplan-Meier. Cumulative incidence functions for salvage interventions were constructed with death as the competing event; group differences were assessed by Gray’s test. Unadjusted Fine-Gray subdistribution hazard regression (reference: 1 lesion) was performed for each salvage endpoint with lesion burden as a three-level categorical variable.

For OS, multivariable Cox proportional-hazards reg­ression was performed with the 5-10 lesion group as ­reference. Covariates were: age (<70 vs ≥70 years), sex, primary cancer histology (NSCLC, breast, melanoma, GI, kidney, SCLC, gynecologic, prostate, thyroid, head and neck, sarcoma, bladder, other), fractionation (single vs multi-fraction), postoperative SRS, prior WBRT, prior craniotomy, treatment era (2017-2019, 2020-2022, 2023-2026), extracranial metastatic disease, peri-index active CNS-directed systemic therapy, KPS (≥90, 70-80, <70), and largest treated lesion diameter (≤20 mm vs >20 mm; resection-cavity diameter for postoperative cases). Model fit was assessed by the concordance statistic. Proportional-hazards assumptions were tested by Schoenfeld residuals. A fully adjusted Fine-Gray subdistribution-hazard model was constructed with the same covariates as the primary Cox model.

Sensitivity analyses included: (1) a stratified Cox model incorporating covariates with time-varying effects as strata; (2) restricted cubic spline modeling of continuous lesion count (df 3-5); (3) bootstrap resampling (R = 500) for the >20 lesion HR; (4) landmark analyses at 6 and 12 months; and (5) a post hoc non-inferiority analysis evaluating whether OS in the 11-20 and >20 groups was non-inferior to the 5-10 reference, using a margin of HR 1.30 (matching the margin pre-specified in JLGK0901^6^ and used subsequently by Nagai et al^10^) and one-sided α = 0.025 (upper bound of two-sided 95% CI < margin).

## Results

### Cohort Characteristics

A total of 3893 consecutive patients were included (Table 1; Supplementary Table S1). Median age was 65 years (range 18-99); 58% were female; among patients with documented data, KPS was ≥70 in 94% (2450 documented; 59% of the full cohort, 37% undocumented); median follow-up by reverse Kaplan-Meier was 41.2 months. Histologies spanned 13 categories, including the more commonly represented non-small cell lung cancer (NSCLC) (33%), breast (16%), GI (10%), and melanoma (9%), and less often represented cancers such as kidney, gynecologic, prostate, thyroid, head and neck, sarcoma, and bladder. Most courses were 3-5 fractions (70%); 18% were postoperative; the largest treated lesion was ≤20 mm in 74% of courses with a documented diameter (56% overall; 25% undocumented). No minimum-follow-up restriction was applied; because OS is measured from end of the index course with censoring at last vital-status contact, recently treated patients contribute valid survival time, and only 96 patients (2.5%) had <3 months between the index course and database lock.

The median number of treated lesions was 2; 77% of index courses targeted 1-4 lesions, while 901 (23%) targeted ≥5, including 206 with 11-20 and 41 with >20. High-burden groups were over-represented in the most recent era: 60% of 11-20 patients and 80% of >20 patients were treated in 2023-2026, versus 7% and 2% in 2017-2019. The 3 most common regimens were 27 Gy in 3 fractions (38%), 30 Gy in 5 fractions (28%), and 21 Gy in 1 fraction (27%).

### Overall Survival

Median OS in the full cohort was 12.2 months (95% CI, 11.4-12.9). OS declined significantly between the 1, 2-4, and 5-10 lesion groups but was similar between the 5-10, 11-20, and >20 lesion groups (log-rank *P* < .001; Figure 1A, Table 2). Median OS was 16.2 months (95% CI, 14.9-17.9) for 1 lesion, 11.0 (9.9-12.0) for 2-4, 8.2 (7.2-9.5) for 5-10, 8.7 (6.7-11.5) for 11-20, and 6.5 (4.6-14.6) for >20.

**Figure 1.**
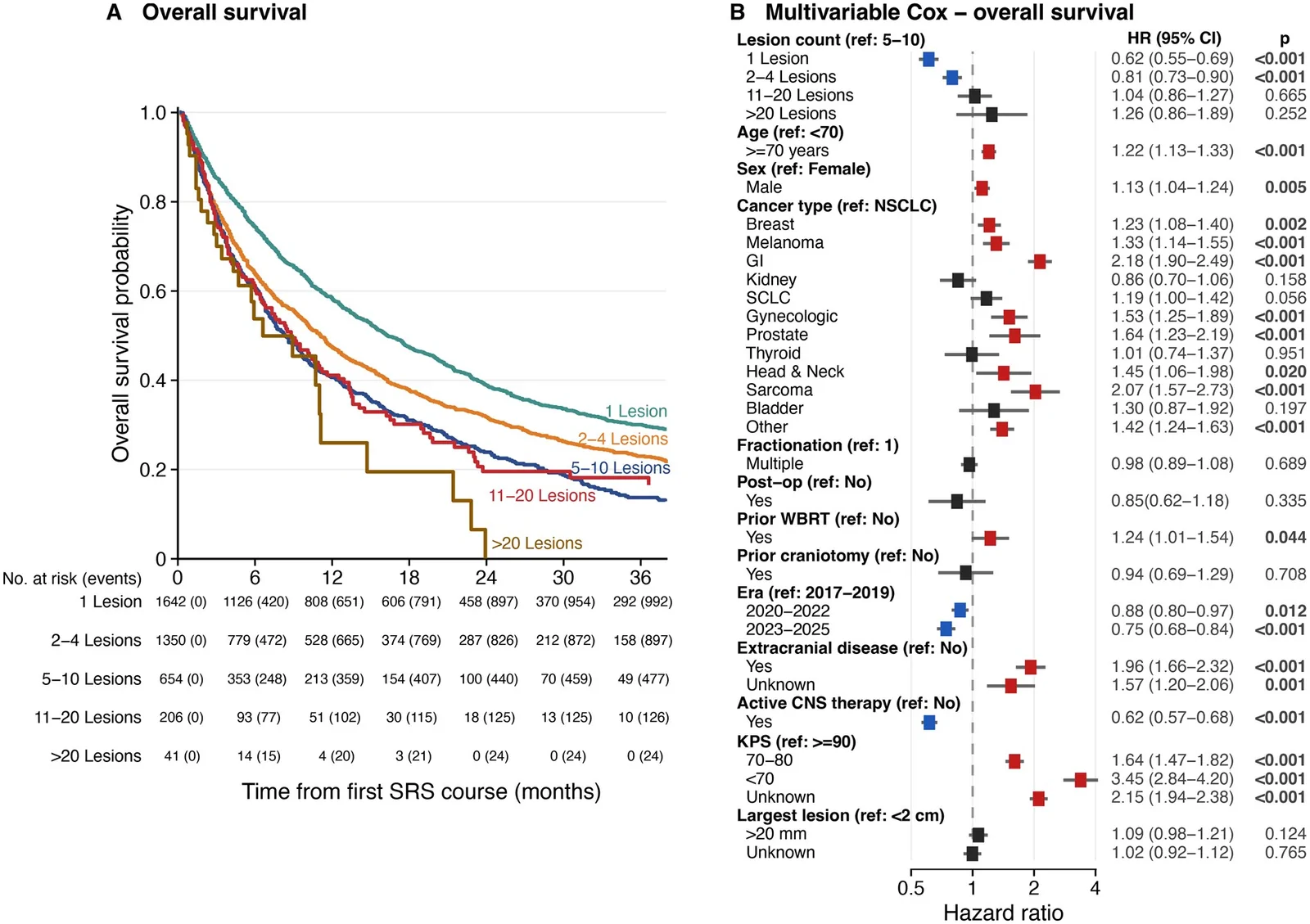
(A) Overall survival by intracranial lesion count. Kaplan-Meier estimates stratified by lesion count at first SRS course (1, 2-4, 5-10, 11-20, >20 lesions; log-rank *P* < .001). Numbers at risk and cumulative event counts are displayed below the x-axis. (B) Multivariable Cox proportional hazards forest plot. Adjusted hazard ratios (HR) with 95% confidence intervals from the full model; reference group: 5-10 lesions.

**Table 2.** Overall survival outcomes and adjusted hazard ratios by lesion burden group

Group	*N*	Median OS (95% CI), months	Events/At Risk	MVA HR (95% CI)a	*P*
1 Lesion	1641	16.2 (14.9-17.9)	1079/1641	0.62 (0.55-0.69)	<.001
2-4 Lesions	1351	11.0 (9.9-12.0)	977/1351	0.81 (0.73-0.90)	<.001
**5-10 Lesions**	**654**	**8.2 (7.2-9.5)**	**501/654**	**Reference**	**—**
11-20 Lesions	206	8.7 (6.7-11.5)	132/206	1.04 (0.86-1.27)	0.665
>20 Lesions	41	6.5 (4.6-14.6)	26/41	1.26 (0.85-1.89)	0.252

a Full multivariable Cox model (*N* = 3893), reference group: 5-10 lesions. Covariates: age (<70 vs ≥70), sex, primary cancer type (PRED), fractionation, postoperative SRS, prior WBRT, prior craniotomy, treatment era, ECM at BM diagnosis, any CNS-active systemic agent. Log-rank *P* (overall) <.001.

On multivariable Cox regression (c-index 0.69) with 5-10 lesions as reference, OS was significantly better for 1 lesion (HR 0.62, 95% CI, 0.55-0.69; *P* < .001) and 2-4 lesions (HR 0.81, 0.73-0.90; *P* < .001) (Figure 1B; Table 2; Supplementary Table S2). Neither the 11-20 group (HR 1.04, 0.86-1.27; *P* = .665) nor the >20 group (HR 1.26, 0.85-1.89; *P* = .252) differed significantly from the 5-10 reference. Post hoc non-inferiority testing (margin HR 1.30, one-sided α = 0.025) confirmed non-inferior OS for 11-20 (upper 95% CI, 1.27 < 1.30; *P* < .001); the >20 group was underpowered to establish non-inferiority (*n* = 41, *P* = .20) and showed a numerically lower median OS.

On restricted cubic spline modeling (Figure 2), the OS hazard rose sharply between 1 and approximately 3 lesions and flattened beyond 5, reinforcing that a patient with ∼15 lesions are prognostically similar to one with ∼7. Landmark analyses at 6 and 12 months, a stratified sensitivity model, and bootstrap resampling for the >20 lesion HR all yielded estimates concordant with the primary analysis (Supplementary Tables S3 and S4).

**Figure 2.**
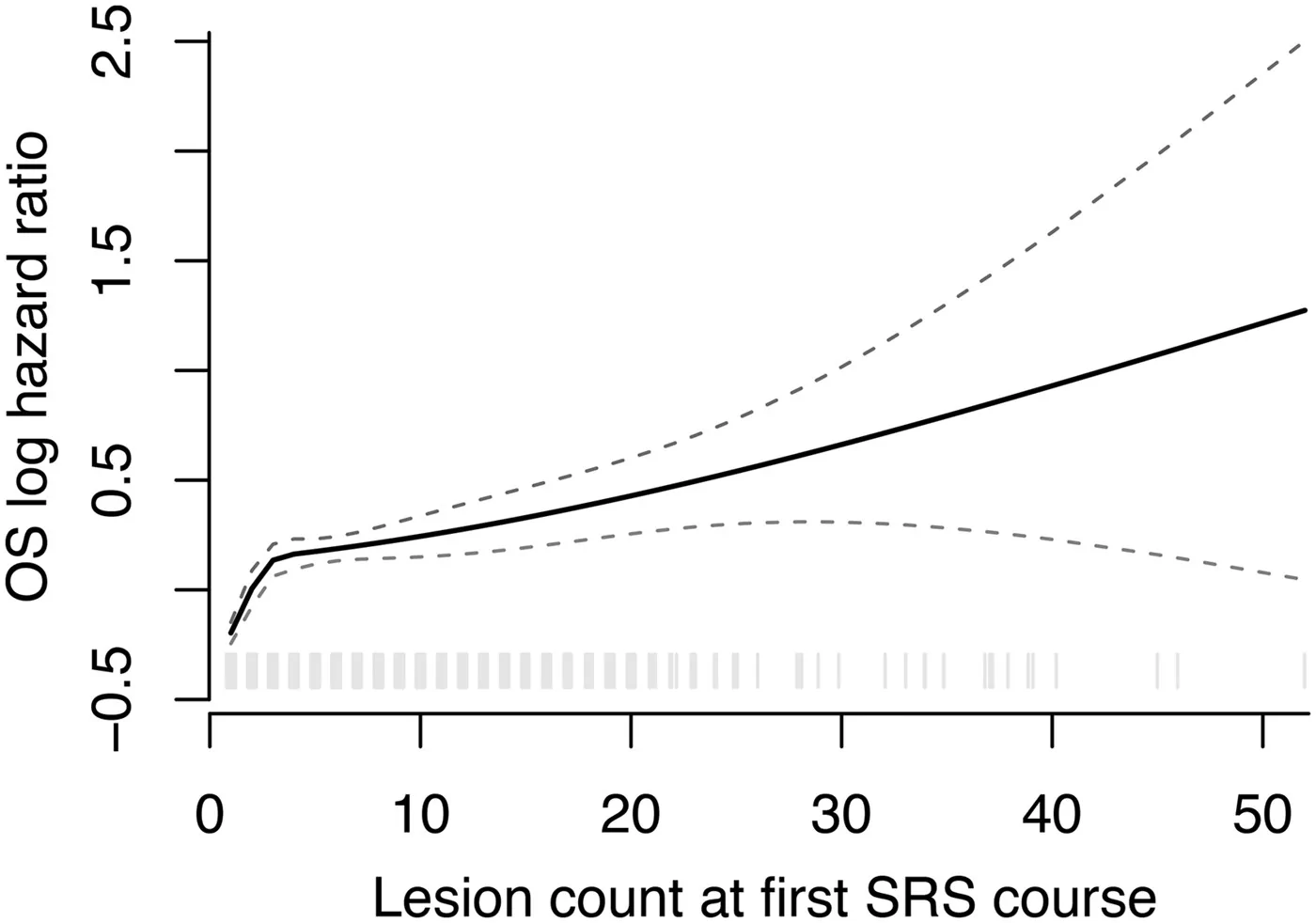
Restricted cubic spline of the log hazard ratio for overall survival as a function of continuous treated lesion count, from the multivariable Cox model. Shaded region represents the 95% confidence band. The reference lesion count is set at the median. Rug marks along the x-axis denote the distribution of individual SRS courses.

Beyond lesion count, several covariates contributed independently to OS (Figure 1B; Supplementary Table S2). As expected, age and KPS were strongly prognostic: relative to KPS ≥90, KPS 70-80 (HR 1.64, 1.47-1.82), and KPS <70 (HR 3.45, 2.84-4.20) had progressively worse OS. Median OS in patients ≥70 years was 10.0 months (95% CI, 9.2-10.9) versus 13.3 (12.4-14.7) in patients <70 (HR 1.22, 1.13-1.33; log-rank *P* < .001). Extracranial metastases at brain metastasis diagnosis nearly doubled the mortality hazard (HR 1.96, 1.66-2.32; *P* < .001). Peri SRS CNS-active systemic therapy was independently associated with substantially improved OS (HR 0.62, 0.57-0.68; *P* < .001), and treatment in 2023-2026 with an approximately 25% reduction in adjusted mortality hazard versus 2017-2019 (HR 0.75, 0.68-0.84; *P* < .001).

Among cancer types (reference: NSCLC), breast (HR 1.23, 1.08-1.40) and melanoma (HR 1.33, 1.14-1.55) were associated with modestly worse OS, while GI cancers carried the ­highest adjusted hazard (HR 2.18, 1.90-2.49). Kidney cancer trended toward more favorable survival (HR 0.86, 0.70-1.06), and SCLC was associated with marginally worse OS (HR 1.19, 1.00-1.42). Several less commonly reported histologies showed significantly worse OS, including gynecologic (HR 1.54, 1.25-1.89), prostate (HR 1.64, 1.23-2.19), sarcoma (HR 2.07, 1.57-2.73), and head and neck primaries (HR 1.45, 1.06-1.98).

Long-term survival (≥30 months) was achieved by a ­substantial fraction of the cohort. Among patients with ≤20 lesions and adequate follow-up (*N* = 3125), 21.7% (*n* = 677) survived ≥30 months, with rates of 27.7%, 19.9%, 13.5%, and 9.9% across the 1, 2-4, 5-10, and 11-20 lesion groups, respectively (Cochran-Armitage trend test χ²=62.3, *P* < .001; ordinal-logistic OR per increment 0.65, 95% CI, 0.58-0.72, *P* < .001). Notably, 87 of 677 long-term survivors (12.9%) had been treated for 5-20 lesions, demonstrating that long-term survival is achievable across this spectrum of treated ­burden, not only in patients with limited disease. No patient with >20 lesions reached the 24-month landmark. On adjusted logistic regression, long-term survivorship was independently associated with lower lesion burden (1 lesion OR 2.15, 1.59-2.93; 2-4 OR 1.61, 1.18-2.20 versus 5-10), younger age, higher KPS, absence of extracranial metastases, and CNS-active systemic therapy; GI primaries (OR 0.28) and SCLC (OR 0.46) were significantly less likely to be long-term survivors (Supplementary Table S5).

### Salvage Treatment Patterns

Cumulative incidences of salvage interventions are shown in Figure 3 and Table 3. Although 70% of patients underwent only a single SRS course, additional SRS was common across all groups, with 12-month cumulative incidences of 22.5%, 27.8%, and 30.2% for the 1, 2-4, and ≥5 lesion groups, respectively (Gray’s *P* < .001). On Fine-Gray regression, higher initial lesion burden modestly increased the rate of additional SRS (sHR 1.21 for 2-4 lesions; sHR 1.26 for ≥5 lesions versus 1 lesion; Supplementary Table S6). The median interval from index to second SRS shortened with increasing initial burden: 6.5 months (IQR 3.4-12.4) for 1 lesion, 5.3 (2.8-10.0) for 2-4 lesions, and 4.5 (2.5-8.0) for ≥5 lesions (log-rank *P* < .001), indicating faster intracranial progression in higher-burden patients.

**Figure 3.**
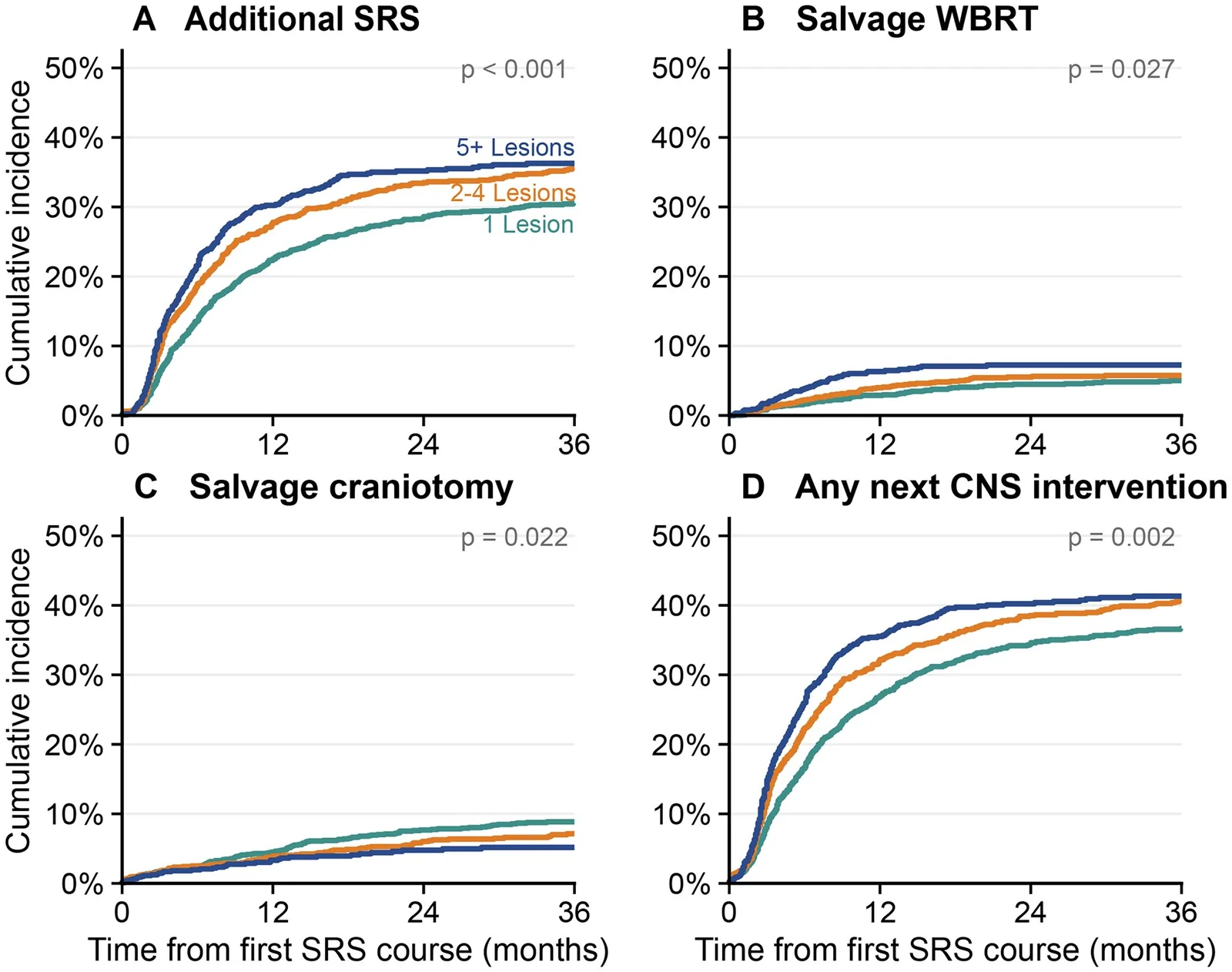
Cumulative incidence of salvage interventions by lesion burden group (1, 2-4, and ≥5 lesions). (A) SRS. (B) WBRT. (C) Craniotomy. (D) Any next CNS intervention, a composite of the first of additional SRS, salvage WBRT, or salvage craniotomy. Competing-risk analysis with death as the competing event. *P*-values from Gray’s test.

**Table 3.** Cumulative incidence of salvage interventions at 6, 12, and 24 months

Endpoint	Lesion Group	6-month CI (%)	12-month CI (%)	24-month CI (%)	Gray’s *P*
Salvage Craniotomy	1 Lesion	2.4	4.5	7.7	.031
	2-4 Lesions	2.5	3.9	6.0	
	5+ Lesions	1.9	3.3	4.8	
Salvage SRS	1 Lesion	13.7	22.5	28.6	<.001
	2-4 Lesions	18.9	27.8	33.5	
	5+ Lesions	21.6	30.2	35.2	
Salvage WBRT	1 Lesion	1.7	2.9	4.5	.023
	2-4 Lesions	2.2	4.0	5.5	
	5+ Lesions	3.9	6.3	7.3	
Any next CNS intervention	1 Lesion	16.7	27.0	34.6	.002
	2-4 Lesions	22.4	32.2	38.5	
	5+ Lesions	26.0	35.5	40.2	

Cumulative incidence estimated by the Fine-Gray competing-risk method; death treated as a competing event. Gray’s test *P*-value for overall between-group difference. Lesion groups: 1, 2-4, ≥5 lesions.

Among patients without prior WBRT, salvage WBRT was uncommon across the full range of treated intracranial burden, with more than 90% of patients receiving none: 12-month cumulative incidences were 2.9%, 4.0%, and 6.3% for the 1, 2-4, and ≥5 lesion groups (Gray’s *P* = .027; Figure 3B; Table 3). On unadjusted Fine-Gray regression, the ≥5 lesion group carried a significantly higher subdistribution hazard versus 1 lesion (sHR 1.55, 1.10-2.19; *P* = .012; Supplementary Table S6).

In a fully adjusted Fine-Gray subdistribution-hazard model among patients without prior WBRT (*N* = 3774; Supplementary Table S7), the ≥5 lesion group retained a significantly higher hazard for WBRT (sHR 1.67, 1.15-2.41; *P* = .007), while the 2-4 group did not differ significantly (sHR 1.21, 0.86-1.70). Among cancer types (reference: NSCLC), breast (sHR 1.86, 1.24-2.79; *P* = .003) and SCLC (sHR 2.58, 1.49-4.46; *P* < .001) were associated with a higher adjusted hazard of WBRT, whereas melanoma (sHR 0.52, 0.26-1.02; *P* = .058) and GI (sHR 0.47, 0.23-0.96; *P* = .040) were borderline or significantly lower (Supplementary Table S8). Age ≥70 was associated with a substantially lower adjusted hazard of WBRT (sHR 0.50, 0.35-0.72; *P* < .001), likely reflecting both competing mortality and clinician propensity to defer WBRT in older patients.

WBRT cumulative incidence decreased over time: compared to 2017-2019, the subdistribution hazard was lower for 2020-2022 (sHR 0.76, 0.54-1.07; *P* = .120) and 2023-2026 (sHR 0.63, 0.44-0.91; *P* = .013). 12-month WBRT incidence fell from 5.4% in 2017-2019 to 3.5% in 2020-2022 and 3.7% in 2023-2026; corresponding 24-month rates were 6.7%, 5.1%, and 4.9% (Supplementary Table S9). KPS, extracranial metastases, CNS-active systemic therapy, fractionation, lesion diameter, and sex were not significantly associated with the adjusted hazard of WBRT in the adjusted model.

To characterize salvage patterns after the second SRS course, we re-indexed follow-up from the date of the second course among the 1208 patients who received ≥2 courses, with death as the competing event. The 12- and 24-month cumulative incidences of a third salvage SRS course were 35.5% and 41.7%, respectively, approximately six-fold higher than the 5.9% and 6.7% incidences for salvage WBRT. The 24-month salvage SRS cumulative incidence (42%) approximated the competing mortality incidence (∼42%), indicating that, after a second SRS course, patients were roughly equally likely to undergo another SRS course or experience mortality, with salvage WBRT remaining uncommon throughout.

Salvage craniotomy showed an inverse trend with increasing lesion burden (Table 3): 12-month rates were 4.5% (1 lesion), 3.9% (2-4 lesions), and 3.3% (≥5 lesions; Gray’s *P* = .022). Fine-Gray regression confirmed a significantly lower craniotomy rate in the ≥5 lesion group (sHR 0.59, 0.41-0.83; *P* = .003).

Considering any next CNS intervention as a single composite endpoint (the first of additional SRS, salvage WBRT, or salvage craniotomy, with death as the competing event), the 24-month cumulative incidence rose modestly with lesion burden: 34.6%, 38.5%, and 40.2% for the 1, 2-4, and ≥5 lesion groups, respectively (Gray’s *P* = .002; adjusted subdistribution hazard 1.23, 95% CI, 1.07-1.42 for ≥5 versus 1 lesion; Figure 3D; Table 3; Supplementary Table S10). This indicates that higher-burden patients experienced more clinically actioned intracranial progression, but that such progression was captured and managed predominantly with focal salvage rather than WBRT.

## Discussion

In this contemporary registry of 3893 patients treated with linac-based SRS for brain metastases across the 7 sites of an NCI-designated multi-site cancer care network, the largest such cohort to date and the first to span the full lesion-burden spectrum encountered in modern practice, we addressed 2 principal questions: whether OS differs between lesion-burden groups, and how often salvage WBRT is required across the spectrum of intracranial burden.

First, OS was significantly better for patients with 1 or 2-4 lesions but similar between patients with 5-10, 11-20, and >20 lesions. Median OS was 16.2 months for a single lesion, 11.0 for 2-4 lesions, and 8.2 for 5-10 lesions, with no significant decrement for 11-20 (median OS 8.7 months) or >20 (6.5 months) compared with the 5-10 reference. Non-inferiority testing formally established equivalent OS for the 11-20 group versus the 5-10 reference (margin HR 1.30, *P* < .001), a margin matching JLGK0901 and closely paralleling its landmark result (HR 1.06, 0.90-1.26). The >20 lesion group did not meet formal non-inferiority (*n* = 41, *P* = .20), and although its median OS was numerically shorter, the small sample size leaves us underpowered to determine whether OS in this subgroup is truly lower or equivalent to the 5-10 reference. While a plateau is observed up to 20 lesions, further research is required to determine whether a true prognostic drop-off occurs beyond 20 lesions. Restricted cubic spline modeling reinforced this pattern: the hazard rose steeply between 1 and approximately 3 lesions before flattening, such that a patient with ∼15 lesions were prognostically similar to one with ∼7. Future characterization of patients with >20 lesions will be essential to refine SRS selection in this rapidly emerging clinical category.

These findings are concordant with a growing body of evidence supporting extension of SRS to higher lesion counts. The JLGK0901 prospective observational study established equivalent OS for 2-4 versus 5-10 Gamma Knife-treated lesions, with no difference in neurocognitive maintenance or complications,^6^^,^^11^ and a subsequent extension demonstrated only marginal differences for 11-20 lesions.^12^ A multi-institutional analysis of 2089 patients similarly found equivalent survival between the 2-4 and 5-15 groups,^13^ and a propensity-matched analysis of 1043 patients reported equivalent neurological death and functional outcomes when SRS was extended beyond JLGK0901 criteria.^14^ The largest prior cohort (5750 patients) reported a stepwise ∼4% increase in hazard per increment of 6-7 lesions,^15^ but pre-dated the immunotherapy era and used a linear model that does not capture the non-linear plateau we observed. Our study extends this evidence base as the largest linac-based cohort reported, spans the contemporary CNS-penetrant systemic-therapy era (2017-2026), and reaches into the >20 lesion category for which prior data are sparse. In contrast to JLGK0901, in which 2-4 and 5-10 groups had equivalent OS, we observed significantly better OS in the 2-4 group, suggesting that equivalence in that selected prospective cohort, which was mostly NSCLC, may not generalize to unselected real-world populations. A recent randomized trial established equivalent OS and superior quality of life for SRS versus hippocampal-avoidance WBRT in 5-20 lesions^7^; our cohort includes 860 patients in this range and an additional 41 beyond it. The few prior linac-based reports have shown comparable OS for 5-10 versus 2-4 lesions,^16^ favorable outcomes for 5-15 lesions in single-center series,^17^ and prospective outcomes from single-isocenter dynamic conformal arc SRS,^18^ and our findings substantially extend that linac-specific evidence base. The contemporary linac platform, frameless, single-isocenter VMAT, now permits noninvasive, single-session treatment of dozens of brain metastases within a routine clinical appointment, which would be difficult to replicate on frame-based Gamma Knife platforms at comparable burden.

Several covariates carried greater prognostic weight than lesion burden once burden exceeded 5 lesions. Histology-specific differences in OS may partly reflect heterogeneous availability and uptake of CNS-penetrant systemic agents across primary tumor types. Age ≥70 conferred a modestly increased mortality hazard (HR 1.22), but median OS in this group was 10.0 months, closely paralleling the JLGK0901-elderly sub-analysis^19^ and supporting offering SRS to older patients based on full clinical context rather than chronologic age alone. CNS-active systemic therapy was strongly protective (HR 0.62), concordant with recent evidence that escalation to higher-CNS-activity systemic agents after SRS is associated with improved survival.^20^ Treatment in 2023-2026 conferred an approximately 25% reduction in adjusted mortality hazard relative to 2017-2019, most plausibly reflecting the cumulative real-world impact of CNS-penetrant TKIs (including in oncogene-driven NSCLC),^21^ immune checkpoint inhibitors, and HER2-directed antibody-drug conjugates,^22-24^ a benefit difficult to isolate within any single trial. A substantial subset of the cohort achieved long-term survival: of 3125 patients with ≤20 lesions and adequate follow-up, 21.7% (*n* = 677) survived ≥30 months. Notably, 13.5% of patients with 5-10 lesions and 9.9% of patients with 11-20 lesions achieved long-term survival, and 87 of 677 long-term survivors (13%) had been treated for 5-20 lesions, demonstrating that long-term survival was achievable across the full burden spectrum and not the exclusive province of patients with limited intracranial disease.

Second, salvage WBRT was uncommon across the full intracranial-burden spectrum, with more than 90% of patients receiving no salvage WBRT. 12-month cumulative incidences by lesion count ranged from 3% to 6% among patients without prior WBRT, concordant with the 5.1% reported in the SRS arm of a recent SRS vs. WBRT trial^7^ and substantially below the 12%-16% rates of older multi-institutional series.^13^ Lesion burden, breast and SCLC histologies, age, and treatment era were independent predictors of WBRT, consistent with prior competing-risk analyses identifying histology and lesion number as predictors of salvage WBRT,^25^ and with risk-stratification scores describing wide variation in early WBRT incidence based on clinical features.^26^ 12-month incidence dropped from 5.4% in 2017-2019 to 3.5%-3.7% in subsequent eras. Looking beyond the index course, additional SRS was common (12-month cumulative incidence 23%-30%), and the median interval between index and second SRS course shortened with increasing initial burden (6.5 vs 4.5 months; *P* < .001). Even after a second SRS course, patients were far more likely to undergo additional SRS or to die than to receive WBRT, describing an SRS-predominant salvage cascade in which WBRT is deferred or avoided altogether through multiple intracranial recurrences. The higher salvage-craniotomy cumulative incidence among single-lesion patients (12-month 4.5% vs 3.3% for ≥5 lesions; adjusted hazard 0.59) reflects practice rather than biology, as resection is more readily offered when intracranial disease is not diffuse.

Several limitations should be acknowledged. This is a retrospective analysis from a multi-site cancer care network with inherent selection bias: patients with higher lesion counts were likely selected for SRS based on favorable performance status and systemic-disease trajectory, which may inflate higher-burden survival estimates. The >20 group is small, with correspondingly wide confidence intervals. KPS and extracranial metastatic status contained missing values, coded as unknown to preserve the full analytic sample, but introducing the possibility of residual confounding. Our CNS-directed systemic-therapy variable was based on a curated agent list with documented intracranial activity ≥30% and may under-capture peri-SRS exposure. Because salvage interventions reflect institutional practice, they cannot be equated with objective radiographic local failure, and we cannot exclude that some patients managed without WBRT might have benefited from salvage hippocampal-avoidance WBRT. Salvage craniotomy was performed for enlarging or symptomatic lesions in which the differential included tumor progression and radiation necrosis, which our administrative dataset does not reliably distinguish. Total intracranial tumor volume, potentially more biologically relevant than lesion count alone,^27^ was not captured and, together with lesion-level local control, radiation necrosis, and their dosimetric predictors, is the focus of an analysis we are actively developing across the full institutional registry. Institutional culture in our network has historically favored SRS-based management of brain metastases, and WBRT utilization rates therefore likely underestimate those of centers with greater reliance on whole-brain paradigms. Finally, we did not assess neurocognitive outcomes or patient-reported quality of life, which underpin the rationale for SRS over WBRT.^3^^,^^4^^,^^28^

Taken together, our findings argue for moving beyond a binary lesion-count cutoff and toward an individualized SRS candidacy assessment that prioritizes clinical context. In practical terms, this means leading with performance status, the trajectory of extracranial disease, and the availability of CNS-active systemic therapy, with intracranial burden serving as a modifier. By this framework, a patient with strong performance status, controlled extracranial disease, and access to CNS-active systemic therapy is an appropriate SRS candidate even at high lesion counts; conversely, the patient with poor performance status and uncontrolled systemic disease may benefit less from any local intervention regardless of burden. Lesion-count thresholds in current guidelines are best interpreted as practical anchor points rather than fixed boundaries. In summary, OS following linac-based SRS was similar between patients with 5-10, 11-20, and >20 treated lesions, salvage WBRT was uncommon, with more than 90% of patients receiving none, regardless of lesion number, and treatment era, histology, performance status, and access to CNS-active systemic therapy emerged as stronger determinants of outcome than lesion count once burden exceeded 5 lesions. Current guidelines acknowledge that the definition of “limited” brain metastases is evolving and that no specific lesion-count threshold can be recommended.^8^^,^^29^^,^^30^ These findings support an individualized, SRS-first strategy guided by clinical factors and institutional expertise rather than a fixed lesion-count threshold.

## Conclusions

In this largest contemporary multi-site linac-based SRS registry to date, OS was similar between patients with 5-10, 11-20, and >20 treated lesions, with formal non-inferiority established for the 11-20 group versus the 5-10 reference; the >20 group was underpowered to determine whether OS is truly lower or equivalent. Long-term survival (≥30 months) was achieved by 22% of the cohort, with 13% of long-term survivors having been treated for 5-20 lesions. Although absolute salvage-WBRT rates were low across all groups (24-month cumulative incidence 4.5%-7.3%), the risk increased significantly with lesion burden (≥5 vs 1 lesion: adjusted subdistribution hazard 1.67, 95% CI, 1.15-2.41); even after a second SRS course, recurrent intracranial disease was preferentially retreated with focal therapy rather than WBRT. Treatment era, histology, performance status, and access to CNS-active systemic therapy emerged as stronger determinants of outcome than lesion count once burden exceeded 5 lesions. These findings support an individualized, SRS-first strategy guided by clinical factors and institutional expertise rather than a fixed lesion-count threshold.

## Supplementary Material

vdag242_Supplementary_Data

## Data Availability

De-identified patient-level data and analytic code supporting this study are available upon reasonable request to the corresponding author, subject to institutional data use agreement.
