# Supplementary material for: Linac-based stereotactic radiosurgery for brain metastases: Survival and salvage treatment in a contemporary cohort of 3893 patients
Source: Neurooncol Adv. 2026 Sep 16;8(1):vdag242. doi: 10.1093/noajnl/vdag242 (PMC13641822; doi:10.1093/noajnl/vdag242)
Supplement: Supplementary file 1 [file vdag242_supplementary_data.docx]

**Supplementary Material**

**Linac-Based Stereotactic Radiosurgery for Brain Metastases: Survival and Salvage Treatment in a Contemporary Cohort of 3,893 Patients**

**Corresponding Author:**
Luke R.G. Pike, MD, DPhil
Memorial Sloan Kettering Cancer Center
1275 York Avenue, New York, NY 10065
Phone: 212-639-8157 | Email: pikel@mskcc.org

Table S1. Patient and treatment characteristics by lesion count group

|  | | | **Overall (N=3,893)** | | | **Lesion count group at first SRS course** | | |
| --- | --- | --- | --- | --- | --- | --- | --- | --- |
| **Characteristic** | **Overall** | **1 Lesion** | | **2–4 Lesions** | **5–10 Lesions** | | **11–20 Lesions** | **>20 Lesions** |
| **N** | **3893** | **1641** | | **1351** | **654** | | **206** | **41** |
| Age at SRS, median (IQR), years | 65.0 (55.0–73.0) | 65.0 (56.0–73.0) | | 65.0 (55.0–73.0) | 64.0 (55.0–71.0) | | 63.0 (54.0–70.8) | 59.0 (55.0–73.0) |
| **Sex** |  |  | |  |  | |  |  |
| Female | 2272 (58.4%) | 918 (55.9%) | | 791 (58.5%) | 401 (61.3%) | | 131 (63.6%) | 31 (75.6%) |
| Male | 1621 (41.6%) | 723 (44.1%) | | 560 (41.5%) | 253 (38.7%) | | 75 (36.4%) | 10 (24.4%) |
| **Primary cancer type (AI PRED)** |  |  | |  |  | |  |  |
| NSCLC | 1301 (33.4%) | 529 (32.2%) | | 464 (34.3%) | 233 (35.6%) | | 69 (33.5%) | 6 (14.6%) |
| Breast | 640 (16.4%) | 252 (15.4%) | | 207 (15.3%) | 113 (17.3%) | | 52 (25.2%) | 16 (39.0%) |
| Melanoma | 342 (8.8%) | 119 (7.3%) | | 133 (9.8%) | 67 (10.2%) | | 20 (9.7%) | 3 (7.3%) |
| GI | 375 (9.6%) | 174 (10.6%) | | 131 (9.7%) | 51 (7.8%) | | 15 (7.3%) | 4 (9.8%) |
| Kidney | 176 (4.5%) | 96 (5.9%) | | 52 (3.8%) | 19 (2.9%) | | 7 (3.4%) | 2 (4.9%) |
| SCLC | 209 (5.4%) | 95 (5.8%) | | 68 (5.0%) | 32 (4.9%) | | 11 (5.3%) | 3 (7.3%) |
| Gynecologic | 141 (3.6%) | 63 (3.8%) | | 49 (3.6%) | 26 (4.0%) | | 3 (1.5%) | 0 (0.0%) |
| Prostate | 71 (1.8%) | 36 (2.2%) | | 24 (1.8%) | 7 (1.1%) | | 3 (1.5%) | 1 (2.4%) |
| Thyroid | 67 (1.7%) | 30 (1.8%) | | 23 (1.7%) | 11 (1.7%) | | 2 (1.0%) | 1 (2.4%) |
| Head & Neck | 57 (1.5%) | 20 (1.2%) | | 22 (1.6%) | 11 (1.7%) | | 4 (1.9%) | 0 (0.0%) |
| Sarcoma | 66 (1.7%) | 27 (1.6%) | | 24 (1.8%) | 12 (1.8%) | | 2 (1.0%) | 1 (2.4%) |
| Bladder | 38 (1.0%) | 18 (1.1%) | | 13 (1.0%) | 6 (0.9%) | | 1 (0.5%) | 0 (0.0%) |
| Other | 410 (10.5%) | 182 (11.1%) | | 141 (10.4%) | 66 (10.1%) | | 17 (8.3%) | 4 (9.8%) |
| **Fractionation** |  |  | |  |  | |  |  |
| Single-fraction | 1161 (29.8%) | 550 (33.5%) | | 421 (31.2%) | 153 (23.4%) | | 36 (17.5%) | 1 (2.4%) |
| Multi-fraction | 2732 (70.2%) | 1091 (66.5%) | | 930 (68.8%) | 501 (76.6%) | | 170 (82.5%) | 40 (97.6%) |
| **Post-operative SRS** |  |  | |  |  | |  |  |
| No | 3197 (82.1%) | 1286 (78.4%) | | 1119 (82.8%) | 567 (86.7%) | | 187 (90.8%) | 38 (92.7%) |
| Yes | 696 (17.9%) | 355 (21.6%) | | 232 (17.2%) | 87 (13.3%) | | 19 (9.2%) | 3 (7.3%) |
| **Prior WBRT** |  |  | |  |  | |  |  |
| No | 3774 (96.9%) | 1602 (97.6%) | | 1312 (97.1%) | 625 (95.6%) | | 197 (95.6%) | 38 (92.7%) |
| Yes | 119 (3.1%) | 39 (2.4%) | | 39 (2.9%) | 29 (4.4%) | | 9 (4.4%) | 3 (7.3%) |
| **Prior craniotomy** |  |  | |  |  | |  |  |
| No | 3133 (80.5%) | 1257 (76.6%) | | 1095 (81.1%) | 559 (85.5%) | | 184 (89.3%) | 38 (92.7%) |
| Yes | 760 (19.5%) | 384 (23.4%) | | 256 (18.9%) | 95 (14.5%) | | 22 (10.7%) | 3 (7.3%) |
| **Treatment era** |  |  | |  |  | |  |  |
| 2017-2019 | 836 (21.5%) | 377 (23.0%) | | 310 (22.9%) | 133 (20.3%) | | 15 (7.3%) | 1 (2.4%) |
| 2020-2022 | 1323 (34.0%) | 587 (35.8%) | | 442 (32.7%) | 219 (33.5%) | | 68 (33.0%) | 7 (17.1%) |
| 2023-2026 | 1734 (44.5%) | 677 (41.3%) | | 599 (44.3%) | 302 (46.2%) | | 123 (59.7%) | 33 (80.5%) |
| **ECM at BM diagnosis** |  |  | |  |  | |  |  |
| No | 308 (7.9%) | 181 (11.0%) | | 90 (6.7%) | 29 (4.4%) | | 8 (3.9%) | 0 (0.0%) |
| Yes | 3450 (88.6%) | 1398 (85.2%) | | 1219 (90.2%) | 601 (91.9%) | | 193 (93.7%) | 39 (95.1%) |
| Unknown | 135 (3.5%) | 62 (3.8%) | | 42 (3.1%) | 24 (3.7%) | | 5 (2.4%) | 2 (4.9%) |
| **Any CNS-active systemic agent** |  |  | |  |  | |  |  |
| No | 2592 (66.6%) | 1111 (67.7%) | | 903 (66.8%) | 421 (64.4%) | | 131 (63.6%) | 26 (63.4%) |
| Yes | 1301 (33.4%) | 530 (32.3%) | | 448 (33.2%) | 233 (35.6%) | | 75 (36.4%) | 15 (36.6%) |
| **Chemotherapy** |  |  | |  |  | |  |  |
| No | 3491 (89.7%) | 1478 (90.1%) | | 1220 (90.3%) | 584 (89.3%) | | 177 (85.9%) | 32 (78.0%) |
| Yes | 402 (10.3%) | 163 (9.9%) | | 131 (9.7%) | 70 (10.7%) | | 29 (14.1%) | 9 (22.0%) |
| **Immunotherapy** |  |  | |  |  | |  |  |
| No | 3562 (91.5%) | 1497 (91.2%) | | 1238 (91.6%) | 595 (91.0%) | | 193 (93.7%) | 39 (95.1%) |
| Yes | 331 (8.5%) | 144 (8.8%) | | 113 (8.4%) | 59 (9.0%) | | 13 (6.3%) | 2 (4.9%) |
| **Targeted** |  |  | |  |  | |  |  |
| No | 3250 (83.5%) | 1381 (84.2%) | | 1122 (83.0%) | 538 (82.3%) | | 173 (84.0%) | 36 (87.8%) |
| Yes | 643 (16.5%) | 260 (15.8%) | | 229 (17.0%) | 116 (17.7%) | | 33 (16.0%) | 5 (12.2%) |
| **KPS (coalesced)** |  |  | |  |  | |  |  |
| ≥90 | 1120 (28.8%) | 528 (32.2%) | | 370 (27.4%) | 156 (23.9%) | | 56 (27.2%) | 10 (24.4%) |
| 70-80 | 1186 (30.5%) | 509 (31.0%) | | 406 (30.1%) | 203 (31.0%) | | 58 (28.2%) | 10 (24.4%) |
| <70 | 144 (3.7%) | 55 (3.4%) | | 51 (3.8%) | 28 (4.3%) | | 6 (2.9%) | 4 (9.8%) |
| Unknown | 1443 (37.1%) | 549 (33.5%) | | 524 (38.8%) | 267 (40.8%) | | 86 (41.7%) | 17 (41.5%) |
| **Largest lesion diameter** |  |  | |  |  | |  |  |
| ≤20 mm | 2162 (55.5%) | 780 (47.5%) | | 821 (60.8%) | 403 (61.6%) | | 133 (64.6%) | 25 (61.0%) |
| >20 mm | 754 (19.4%) | 255 (15.5%) | | 285 (21.1%) | 162 (24.8%) | | 42 (20.4%) | 10 (24.4%) |
| Unknown | 977 (25.1%) | 606 (36.9%) | | 245 (18.1%) | 89 (13.6%) | | 31 (15.0%) | 6 (14.6%) |

Table S2. Full multivariable Cox proportional hazards model for overall survival

| **Section** | **Covariate** | **HR (95% CI)** | **p** |
| --- | --- | --- | --- |
| Lesion count (ref: 5–10 lesions) | 1 Lesion | 0.62 (0.55–0.69) | <0.001 |
|  | 2–4 Lesions | 0.81 (0.73–0.90) | <0.001 |
|  | 11–20 Lesions | 1.04 (0.86–1.27) | 0.665 |
|  | >20 Lesions | 1.26 (0.85–1.89) | 0.252 |
| Age (ref: <70) | Age ≥70 years | 1.22 (1.13–1.33) | <0.001 |
| Sex (ref: Female) | Male sex | 1.13 (1.04–1.24) | 0.005 |
| Cancer type (ref: NSCLC) | Breast | 1.23 (1.08–1.40) | 0.002 |
|  | Melanoma | 1.33 (1.14–1.55) | <0.001 |
|  | GI | 2.18 (1.90–2.49) | <0.001 |
|  | Kidney | 0.86 (0.70–1.06) | 0.158 |
|  | SCLC | 1.19 (1.00–1.42) | 0.056 |
|  | Gynecologic | 1.54 (1.25–1.89) | <0.001 |
|  | Prostate | 1.64 (1.23–2.19) | <0.001 |
|  | Thyroid | 1.01 (0.74–1.37) | 0.951 |
|  | Head & Neck | 1.45 (1.06–1.98) | 0.020 |
|  | Sarcoma | 2.07 (1.57–2.73) | <0.001 |
|  | Bladder | 1.30 (0.87–1.92) | 0.197 |
|  | Other | 1.42 (1.24–1.63) | <0.001 |
| Fractionation (ref: Single-fraction) | Multi-fraction | 0.98 (0.89–1.08) | 0.689 |
| Post-operative SRS (ref: No) | Post-operative SRS: Yes | 0.85 (0.62–1.18) | 0.335 |
| Prior WBRT (ref: No) | Prior WBRT: Yes | 1.24 (1.01–1.54) | 0.044 |
| Prior craniotomy (ref: No) | Prior craniotomy: Yes | 0.94 (0.69–1.29) | 0.708 |
| Treatment era (ref: 2017–2019) | Era 2020–2022 | 0.88 (0.80–0.97) | 0.012 |
|  | Era 2023–2026 | 0.75 (0.68–0.84) | <0.001 |
| ECM at BM diagnosis (ref: No) | ECM at BM diagnosis: Yes | 1.96 (1.66–2.32) | <0.001 |
|  | ECM at BM diagnosis: Unknown | 1.57 (1.20–2.06) | 0.001 |
| CNS-active systemic agent (ref: No) | CNS-active systemic agent: Yes | 0.62 (0.57–0.68) | <0.001 |
| KPS (ref: ≥90) | KPS 70–80 | 1.64 (1.47–1.82) | <0.001 |
|  | KPS <70 | 3.45 (2.84–4.20) | <0.001 |
|  | KPS Unknown | 2.15 (1.94–2.38) | <0.001 |
| Largest lesion diameter (ref: ≤20 mm) | >20 mm | 1.09 (0.98–1.21) | 0.124 |
|  | Unknown | 1.02 (0.92–1.12) | 0.765 |
| N = 3893, events (deaths) = 2715. Efron method for ties. Concordance (C-index) = 0.685. Missing KPS, ECM, and lesion diameter coded as 'Unknown' to preserve full sample size. CNS-active systemic agent derived from raw_medication (no record = No). | | | |

Table S3. Landmark analysis: lesion group hazard ratios at 6- and 12-month landmarks

| **Analysis** | **Lesion Group** | **HR (95% CI)** | **p** |
| --- | --- | --- | --- |
| Full cohort (N=3893, events=2715) | 1 Lesion | 0.62 (0.55–0.69) | <0.001 |
|  | 2-4 Lesions | 0.81 (0.73–0.90) | <0.001 |
|  | 11-20 Lesions | 1.04 (0.86–1.27) | 0.665 |
|  | >20 Lesions | 1.26 (0.85–1.89) | 0.252 |
| 6-month landmark (N=2391, events=1463) | 1 Lesion | 0.63 (0.54–0.73) | <0.001 |
|  | 2-4 Lesions | 0.75 (0.64–0.88) | <0.001 |
|  | 11-20 Lesions | 0.99 (0.74–1.33) | 0.957 |
|  | >20 Lesions | 1.39 (0.70–2.79) | 0.348 |
| 12-month landmark (N=1632, events=890) | 1 Lesion | 0.66 (0.54–0.81) | <0.001 |
|  | 2-4 Lesions | 0.80 (0.65–0.98) | 0.031 |
|  | 11-20 Lesions | 1.01 (0.67–1.54) | 0.949 |
|  | >20 Lesions | 1.86 (0.64–5.39) | 0.256 |
| Reference group: 5–10 lesions. All landmark models include same covariates as full model (including ECM, CNS therapy, KPS, lesion diameter; missing values coded as Unknown). Patients who died or were censored before the landmark were excluded from each landmark analysis. | | | |

Table S4. Primary versus stratified sensitivity Cox model: lesion group hazard ratios

| **Model** | **Lesion Group** | **HR (95% CI)** | **p** |
| --- | --- | --- | --- |
| Primary | 1 Lesion | 0.62 (0.56–0.69) | <0.001 |
|  | 2-4 Lesions | 0.81 (0.72–0.90) | <0.001 |
|  | 11-20 Lesions | 1.04 (0.86–1.27) | 0.665 |
|  | >20 Lesions | 1.26 (0.85–1.89) | 0.252 |
|  | lesion_diam_cat>20 mm | 1.09 (0.98–1.21) | 0.124 |
|  | lesion_diam_catUnknown | 1.01 (0.92–1.12) | 0.765 |
| Stratified (sensitivity) | 1 Lesion | 0.62 (0.56–0.70) | <0.001 |
|  | 2-4 Lesions | 0.81 (0.72–0.90) | <0.001 |
|  | 11-20 Lesions | 1.02 (0.84–1.24) | 0.856 |
|  | >20 Lesions | 1.33 (0.89–2.00) | 0.166 |
|  | lesion_diam_cat>20 mm | 1.08 (0.97–1.20) | 0.181 |
|  | lesion_diam_catUnknown | 1.01 (0.91–1.12) | 0.856 |
| Primary model: standard Cox with all covariates as fixed effects, reference: 5–10 lesions. Stratified sensitivity model: variables violating PH incorporated as strata (baseline hazard allowed to vary by stratum). | | | |

Table S5. Adjusted logistic regression for long-term survivorship (OS ≥30 months) among patients with ≤20 lesions

| **Section** | **Covariate** | **OR (95% CI)** | **p** |
| --- | --- | --- | --- |
| Lesion count (ref: 5–10 lesions) | 1 Lesion | 2.15 (1.59-2.93) | <0.001 |
| Lesion count (ref: 5–10 lesions) | 2–4 Lesions | 1.61 (1.18-2.20) | 0.003 |
| Lesion count (ref: 5–10 lesions) | 11–20 Lesions | 0.76 (0.39-1.39) | 0.391 |
| Age (ref: <70) | Age ≥70 years | 0.75 (0.60-0.92) | 0.007 |
| Sex (ref: Female) | Male sex | 0.82 (0.65-1.02) | 0.069 |
| Cancer type (ref: NSCLC) | Breast | 0.73 (0.54-0.98) | 0.036 |
| Cancer type (ref: NSCLC) | Melanoma | 0.81 (0.58-1.13) | 0.226 |
| Cancer type (ref: NSCLC) | GI | 0.28 (0.18-0.43) | <0.001 |
| Cancer type (ref: NSCLC) | Kidney | 1.39 (0.90-2.12) | 0.128 |
| Cancer type (ref: NSCLC) | SCLC | 0.46 (0.25-0.78) | 0.006 |
| Cancer type (ref: NSCLC) | Gynecologic | 0.45 (0.25-0.77) | 0.005 |
| Cancer type (ref: NSCLC) | Prostate | 0.63 (0.27-1.36) | 0.263 |
| Cancer type (ref: NSCLC) | Thyroid | 1.07 (0.54-2.04) | 0.851 |
| Cancer type (ref: NSCLC) | Head & Neck | 0.56 (0.23-1.24) | 0.176 |
| Cancer type (ref: NSCLC) | Sarcoma | 0.57 (0.22-1.25) | 0.189 |
| Cancer type (ref: NSCLC) | Bladder | 0.52 (0.12-1.61) | 0.314 |
| Cancer type (ref: NSCLC) | Other | 0.57 (0.39-0.81) | 0.002 |
| Fractionation (ref: Single-fraction) | Multi-fraction | 0.93 (0.73-1.18) | 0.550 |
| Post-operative SRS (ref: No) | Post-operative SRS: Yes | 1.28 (0.61-2.89) | 0.529 |
| Prior WBRT (ref: No) | Prior WBRT: Yes | 0.57 (0.30-1.04) | 0.079 |
| Prior craniotomy (ref: No) | Prior craniotomy: Yes | 1.13 (0.52-2.33) | 0.744 |
| Treatment era (ref: 2017–2019) | Era 2020–2022 | 1.35 (1.08-1.68) | 0.008 |
| Treatment era (ref: 2017–2019) | Era 2023–2026 | 0.50 (0.38-0.65) | <0.001 |
| ECM at BM diagnosis (ref: No) | ECM at BM diagnosis: Yes | 0.32 (0.24-0.44) | <0.001 |
| ECM at BM diagnosis (ref: No) | ECM at BM diagnosis: Unknown | 0.52 (0.29-0.91) | 0.025 |
| CNS-active systemic agent (ref: No) | CNS-active systemic agent: Yes | 1.75 (1.42-2.15) | <0.001 |
| KPS (ref: ≥90) | KPS 70–80 | 0.41 (0.33-0.52) | <0.001 |
| KPS (ref: ≥90) | KPS <70 | 0.07 (0.02-0.16) | <0.001 |
| KPS (ref: ≥90) | KPS Unknown | 0.28 (0.22-0.35) | <0.001 |
| Largest lesion diameter (ref: ≤20 mm) | >20 mm | 1.21 (0.91-1.59) | 0.184 |
| Largest lesion diameter (ref: ≤20 mm) | Unknown | 1.24 (0.98-1.58) | 0.077 |
| Multivariable logistic regression for the binary outcome of overall survival ≥30 months. Analytic sample restricted to patients with ≤20 lesions and either an event or ≥30 months of follow-up. Reference for lesion count: 5–10 lesions. Missing KPS, ECM, CNS therapy, and lesion diameter coded as 'Unknown' to preserve full sample size. N = 3125; long-term survivors = 677 (21.7%). Note: the treatment-era coefficient is confounded by differential follow-up, patients treated in 2023–2026 who remain alive but have not yet reached 30 months are not evaluable and are excluded, while their early deaths are retained, biasing the recent-era odds ratio downward; it should not be interpreted causally (see the Cox model, Table S2). | | | |

Table S6. Fine-Gray subdistribution hazard ratios for salvage interventions (unadjusted)

| **Endpoint** | **Covariate** | **sHR (95% CI)** | **p** |
| --- | --- | --- | --- |
| Salvage SRS/SRT | 2–4 Lesions (ref: 1 Lesion) | 1.21 (1.06–1.37) | 0.004 |
|  | ≥5 Lesions (ref: 1 Lesion) | 1.26 (1.09–1.46) | 0.002 |
| Salvage WBRT | 2–4 Lesions (ref: 1 Lesion) | 1.18 (0.85–1.63) | 0.320 |
|  | ≥5 Lesions (ref: 1 Lesion) | 1.55 (1.10–2.19) | 0.012 |
| Salvage Craniotomy | 2–4 Lesions (ref: 1 Lesion) | 0.88 (0.68–1.14) | 0.340 |
|  | ≥5 Lesions (ref: 1 Lesion) | 0.59 (0.41–0.83) | 0.003 |
| Fine–Gray subdistribution hazard regression, unadjusted. Reference: 1 lesion. Death treated as competing event. 95% CI computed from standard errors using normal approximation. | | | |

Table S7. Adjusted Fine-Gray subdistribution hazard regression for salvage WBRT

| **Variable** | **sHR (95% CI)** | **p-value** |
| --- | --- | --- |
| Lesion count (ref: 1 Lesion): 2-4 Lesions | 1.21 (0.86–1.70) | 0.280 |
| Lesion count (ref: 1 Lesion): 5+ Lesions | 1.67 (1.15–2.41) | 0.007 |
| Age (ref: <70): ≥70 years | 0.50 (0.35–0.72) | <0.001 |
| Sex (ref: Female): Male | 1.06 (0.74–1.50) | 0.760 |
| Cancer type (ref: NSCLC): Breast | 1.86 (1.24–2.79) | 0.003 |
| Cancer type (ref: NSCLC): Melanoma | 0.52 (0.26–1.02) | 0.056 |
| Cancer type (ref: NSCLC): GI | 0.47 (0.23–0.96) | 0.040 |
| Cancer type (ref: NSCLC): Kidney | 0.54 (0.22–1.34) | 0.180 |
| Cancer type (ref: NSCLC): SCLC | 2.58 (1.49–4.46) | <0.001 |
| Cancer type (ref: NSCLC): Gynecologic | 0.68 (0.27–1.71) | 0.410 |
| Cancer type (ref: NSCLC): Prostate | 0.84 (0.20–3.53) | 0.810 |
| Cancer type (ref: NSCLC): Thyroid | 1.15 (0.41–3.22) | 0.790 |
| Cancer type (ref: NSCLC): Head & Neck | 0.66 (0.16–2.77) | 0.570 |
| Cancer type (ref: NSCLC): Sarcoma | 1.53 (0.61–3.85) | 0.370 |
| Cancer type (ref: NSCLC): Bladder | 2.54 (0.76–8.48) | 0.130 |
| Cancer type (ref: NSCLC): Other | 1.07 (0.62–1.83) | 0.820 |
| Fractionation (ref: Single-fraction): Multi-fraction | 0.99 (0.70–1.40) | 0.930 |
| Post-operative SRS (ref: No): Yes | 0.89 (0.32–2.52) | 0.830 |
| Prior craniotomy (ref: No): Yes | 1.83 (0.67–4.98) | 0.240 |
| Treatment era (ref: 2017-2019): 2020-2022 | 0.76 (0.54–1.07) | 0.120 |
| Treatment era (ref: 2017-2019): 2023-2026 | 0.63 (0.44–0.91) | 0.013 |
| ECM at BM diagnosis (ref: No): Yes | 0.89 (0.55–1.46) | 0.650 |
| ECM at BM diagnosis (ref: No): Unknown | 1.15 (0.52–2.54) | 0.730 |
| Any CNS-active systemic agent (ref: No): Yes | 1.26 (0.94–1.69) | 0.130 |
| KPS (ref: ≥90): 70-80 | 1.15 (0.81–1.64) | 0.430 |
| KPS (ref: ≥90): <70 | 0.49 (0.15–1.63) | 0.250 |
| KPS (ref: ≥90): Unknown | 0.84 (0.58–1.22) | 0.360 |
| Largest lesion diameter (ref: ≤20 mm): >20 mm | 0.88 (0.59–1.29) | 0.510 |
| Largest lesion diameter (ref: ≤20 mm): Unknown | 0.76 (0.52–1.12) | 0.170 |
| Fully adjusted Fine-Gray subdistribution hazard model for receipt of salvage WBRT. Competing event: death. Patients with prior WBRT excluded (N = 3774). Covariates: lesion count, age, sex, cancer type, fractionation, post-operative SRS, prior craniotomy, treatment era, extracranial metastatic disease (ECM), any CNS-active systemic agent (raw_medication), KPS, and largest lesion diameter. Missing KPS, ECM, and lesion diameter coded as "Unknown." | | |

**Table S8. Cumulative incidence of salvage WBRT by primary cancer type**

| **Cancer type** | **6-month CI (%)** | **12-month CI (%)** | **24-month CI (%)** | **sHR (95% CI)** | **p** | **Gray's p** |
| --- | --- | --- | --- | --- | --- | --- |
| NSCLC | 1.9 | 3.2 | 4.7 | Reference (NSCLC) | — | <0.001 |
| Breast | 4.4 | 6.6 | 10.1 | 1.84 (1.23–2.75) | 0.003 |  |
| Melanoma | 1.2 | 2.5 | 3.2 | 0.52 (0.26–1.02) | 0.058 |  |
| GI | 1.7 | 2.3 | 2.6 | 0.52 (0.26–1.04) | 0.066 |  |
| Kidney | 1.2 | 2.5 | 3.2 | 0.54 (0.22–1.35) | 0.190 |  |
| SCLC | 2.0 | 7.5 | 8.9 | 2.54 (1.47–4.40) | <0.001 |  |
| Gynecologic | 0.7 | 3.1 | 3.9 | 0.67 (0.26–1.69) | 0.390 |  |
| Prostate | 1.6 | 3.3 | 3.3 | 0.84 (0.20–3.55) | 0.820 |  |
| Thyroid | 1.5 | 3.1 | 6.7 | 1.16 (0.41–3.23) | 0.780 |  |
| Head & Neck | 3.6 | 3.6 | 3.6 | 0.66 (0.15–2.77) | 0.570 |  |
| Sarcoma | 4.6 | 6.2 | 6.2 | 1.53 (0.61–3.82) | 0.360 |  |
| Bladder | 7.9 | 7.9 | 7.9 | 2.55 (0.77–8.48) | 0.130 |  |
| Other | 2.8 | 4.5 | 4.9 | 1.06 (0.62–1.83) | 0.820 |  |
| Cumulative incidence estimated by Fine–Gray competing-risk method; death treated as competing event. Gray’s test p-value for overall between-group difference. Adjusted subdistribution HRs (sHR) from Fine–Gray regression adjusted for lesion burden, age, sex, fractionation, post-operative SRS, prior WBRT, prior craniotomy, and treatment era; reference: NSCLC. | | | | | | |

**Table S9. Cumulative incidence of salvage WBRT by treatment era**

| **Treatment era** | **6-month CI (%)** | **12-month CI (%)** | **24-month CI (%)** | **Gray's p** |
| --- | --- | --- | --- | --- |
| 2017-2019 | 3.1 | 5.4 | 6.7 | 0.305 |
| 2020-2022 | 2.0 | 3.5 | 5.1 |  |
| 2023-2026 | 2.3 | 3.7 | 4.9 |  |
| Cumulative incidence estimated by Fine–Gray competing-risk method; death treated as competing event. Gray’s test p-value for overall between-era difference. Fine–Gray subdistribution HRs (adjusted): 0.78 (0.55–1.09) (2020–2022 vs 2017–2019, p=0.150); 0.64 (0.45–0.92) (2023–2026 vs 2017–2019, p=0.014). | | | | |

**Table S10. Adjusted Fine–Gray subdistribution hazard regression for the composite next CNS intervention endpoint**

| **Variable** | **sHR (95% CI)** | **p-value** |
| --- | --- | --- |
| Lesion count (ref: 1 Lesion): 2-4 Lesions | 1.19 (1.05-1.34) | 0.005 |
| Lesion count (ref: 1 Lesion): 5+ Lesions | 1.23 (1.07-1.42) | 0.004 |
| Age (ref: <70): ≥70 | 0.76 (0.67-0.86) | <0.001 |
| Sex (ref: Female): Male | 1.01 (0.89-1.14) | 0.870 |
| Cancer type (ref: NSCLC): Breast | 1.03 (0.88-1.21) | 0.710 |
| Cancer type (ref: NSCLC): Melanoma | 1.07 (0.88-1.30) | 0.520 |
| Cancer type (ref: NSCLC): GI | 0.99 (0.80-1.22) | 0.920 |
| Cancer type (ref: NSCLC): Kidney | 1.00 (0.77-1.29) | 0.990 |
| Cancer type (ref: NSCLC): SCLC | 1.61 (1.27-2.04) | <0.001 |
| Cancer type (ref: NSCLC): Gynecologic | 1.09 (0.81-1.48) | 0.560 |
| Cancer type (ref: NSCLC): Prostate | 0.50 (0.27-0.92) | 0.027 |
| Cancer type (ref: NSCLC): Thyroid | 0.81 (0.54-1.23) | 0.330 |
| Cancer type (ref: NSCLC): Head & Neck | 1.36 (0.91-2.04) | 0.130 |
| Cancer type (ref: NSCLC): Sarcoma | 1.37 (0.89-2.11) | 0.160 |
| Cancer type (ref: NSCLC): Bladder | 1.38 (0.80-2.39) | 0.250 |
| Cancer type (ref: NSCLC): Other | 0.89 (0.72-1.10) | 0.270 |
| Fractionation (ref: Single-fraction): Multi-fraction | 0.96 (0.84-1.10) | 0.560 |
| Post-operative SRS (ref: No): Yes | 1.08 (0.70-1.67) | 0.720 |
| Prior WBRT (ref: No): Yes | 1.01 (0.75-1.36) | 0.940 |
| Prior craniotomy (ref: No): Yes | 1.23 (0.80-1.87) | 0.340 |
| Treatment era (ref: 2017-2019): 2020-2022 | 1.19 (1.03-1.37) | 0.020 |
| Treatment era (ref: 2017-2019): 2023-2026 | 1.41 (1.22-1.62) | <0.001 |
| ECM at BM diagnosis (ref: No): Yes | 0.84 (0.69-1.01) | 0.057 |
| ECM at BM diagnosis (ref: No): Unknown | 1.16 (0.84-1.58) | 0.370 |
| Any CNS-active systemic agent (ref: No): Yes | 1.48 (1.32-1.66) | <0.001 |
| KPS (ref: ≥90): 70-80 | 1.05 (0.92-1.19) | 0.490 |
| KPS (ref: ≥90): <70 | 0.56 (0.38-0.83) | 0.004 |
| KPS (ref: ≥90): Unknown | 0.70 (0.61-0.80) | <0.001 |
| Largest lesion diameter (ref: ≤20 mm): >20 mm | 0.93 (0.79-1.08) | 0.340 |
| Largest lesion diameter (ref: ≤20 mm): Unknown | 0.87 (0.76-0.99) | 0.037 |

*Fully adjusted Fine–Gray subdistribution hazard model for the composite endpoint of any next CNS intervention (first of additional SRS/SRT, salvage WBRT, or salvage craniotomy); competing event: death. Reference: 1 lesion. N = 3,893; composite events = 1,411. Missing KPS, ECM, and lesion diameter coded as 'Unknown.' Gray's test p=0.002.*
